# Longevity of Middle East Respiratory Syndrome Coronavirus Antibody Responses in Humans, Saudi Arabia

**DOI:** 10.3201/eid2705.204056

**Published:** 2021-05

**Authors:** Abeer N. Alshukairi, Jincun Zhao, Maha A. Al-Mozaini, Yanqun Wang, Ashraf Dada, Salim A. Baharoon, Sara Alfaraj, Waleed A. Ahmed, Mushira A. Enani, Fatehi E. Elzein, Nazik Eltayeb, Laila Layqah, Aiman El-Saed, Husam A. Bahaudden, Abdul Haseeb, Sherif A. El-Kafrawy, Ahmed M. Hassan, Najlaa A. Siddiq, Ibtihaj Alsharif, Isamel Qushmaq, Esam I. Azhar, Stanley Perlman, Ziad A. Memish

**Affiliations:** King Faisal Specialist Hospital and Research Center, Jeddah, Saudi Arabia (A.N. Alshukairi, A. Dada, N.A. Siddiq, I. Qushmaq);; First Affiliated Hospital of Guangzhou Medical University, Guangzhou, China (J. Zhao, Y. Wang);; King Faisal Specialist Hospital and Research Center, Riyadh, Saudi Arabia (M.A. Al-Moziani, I. Alsharif);; King Abdulaziz Medical City, Riyadh (S.A. Baharoon, A. El-Saed);; Prince Mohammed Bin Abdulaziz Hospital, Riyadh (S. Alfaraj);; Security Forces Hospital, Makkah, Saudi Arabia (W.A. Ahmed);; King Fahad Medical City, Riyadh (M.A. Enani);; Prince Sultan Military Medical City, Riyadh (F.E. Elzein, N. Eltayeb);; King Abdullah International Medical Research Center, Riyadh (L. Layqah);; King Saud bin Abdulaziz University for Health Sciences, Jeddah (H.A. Bahaudden);; Umm Al Qura University, Makkah, Saudi Arabia (A. Haseeb);; King Abdulaziz University, Jeddah (S.A. El-Kafrawy, A.M. Hassan);; King Fahd Medical Research Center, Jeddah (E.F. Azhar);; University of Iowa, Iowa City, Iowa, USA (S. Perlman);; King Saud Medical City, Riyadh (Z.A. Memish);; Al-Faisal University, Riyadh (Z.A. Memish)

**Keywords:** Middle East respiratory syndrome coronavirus, MERS-CoV, coronaviruses, viruses, Middle East respiratory syndrome, MERS, antibody responses, longevity, humans, patients, disease severity, zoonoses, Saudi Arabia

## Abstract

Understanding the immune response to Middle East respiratory syndrome coronavirus (MERS-CoV) is crucial for disease prevention and vaccine development. We studied the antibody responses in 48 human MERS-CoV infection survivors who had variable disease severity in Saudi Arabia. MERS-CoV–specific neutralizing antibodies were detected for 6 years postinfection.

Three novel human coronaviruses have caused different worldwide outbreaks that had variable disease severity and geographic distribution: severe acute respiratory syndrome coronavirus (SARS-CoV) during 2003; Middle East respiratory syndrome (MERS) coronavirus (MERS-CoV) during 2012; and severe acute respiratory syndrome coronavirus 2 (SARS-CoV-2), which caused coronavirus disease starting in 2019 (*1*). Understanding the immune response to coronavirus infections is crucial for vaccine development and disease prevention (*2*). Recurrent MERS-CoV infection has not been described in humans. However, longitudinal studies in seropositive camels detected recurrent infections and intermittent shedding of RNA (*3*).

A limited number of studies have evaluated the longevity of MERS antibody responses. Payne et al. described persistence of MERS-CoV neutralizing antibodies for >34 months postinfection in 6 (86%) of 7 survivors (*4*). Choe et al. showed that patients who had severe disease had robust MERS-CoV neutralizing antibody titers for 1 year, and patients who had mild disease had waning antibody response over time (*5*). We assessed antibody responses in 48 MERS survivors who had variable disease severity and duration <6 years postinfection.

## The Study

We recruited 48 MERS survivors from 5 hospitals in Jeddah and Riyadh, Saudi Arabia. All participants who agreed to participate provided consent. The study was approved by the institutional research boards of the hospitals involved. All MERS cases were diagnosed on the basis of positive reverse transcription PCR results. Disease severity was divided into 3 categories: mild infection (asymptomatic and upper respiratory tract infection), moderate infection (pneumonia not requiring intubation and ventilation), and severe infection (pneumonia requiring intubation and ventilation in the intensive care unit). Blood samples were collected for serologic testing from survivors in various hospitals at a single time point, except for 1 patient (case-patient 45; Table) who provided samples at 4 and 6 years postinfection. On the basis of date of diagnosis, MERS-CoV antibody responses were measured 2–6 years postinfection.

An ELISA was performed for 45/49 samples. Microneutralization assays were performed for 43/49 samples in China and 6/49 samples in Saudi Arabia. A total of 43/49 samples were collected 2–5 years postinfection, and 6/49 samples were collected 6 years postinfection. A commercial MERS-CoV S1ELISA Kit (Euroimmun, https://www.euroimmun.com) was used to measure human IgG titers against the MERS-CoV spike protein as described (*6*). Samples with an optical density >1.1 were considered positive, those <0.8 negative, and those 0.8–1.1 borderline. A MERS-CoV focus reduction neutralization test (modified microneutralization assay) and a MERS-CoV microneutralization test were performed in certified Biosafety Level 3 laboratories in Guangzhou, China, and Jeddah, Saudi Arabia, as described (*7*,*8*). The cutoff value for a positive neutralization assay result was 1:20 (Appendix). We used reference MERS-CoV isolates (GenBank accession nos. EMC/2012 in Guangzhou and KF958702 in Jeddah).

We presented continuous variables as median and interquartile range (IQR). We used Kruskal-Wallis, Mann-Whitney, Jonckheere-Terpstra, Fisher exact, and Gamma tests to study the differences between variables. All p values were 2-tailed, and p values <0.05 were considered significant. We used SPSS Statistics 25.0 (IBM Corp., https://www.ibm.com) for all statistical analyses.

Of 49 specimens, 28 (57.1%) were collected from MERS convalescent patients at 2–3 years postinfection, 12 (24.5%) at 4 years postinfection, and 9 (18.4%) at 5–6 years postinfection. Of 49 specimens, 31 (63.3%) were collected from MERS convalescent patients who had mild disease, 12 (24.5%) from those who had moderate disease, and 6 (12.2%) from those who had severe disease (Table). We found that 38/49 specimens had neutralizing antibodies (median [IQR] titer 45 [29–161]). Of these 38 samples, 12 (31%) were negative by ELISA. Ten of these 12 samples were collected from survivors who had mild illness (Table).

**Table:** Clinical and serologic findings for 48 patients 2–6 y after infection with Middle East respiratory syndrome coronavirus, Saudi Arabia*

Patient ID	Age, y/sex	Time, y between serologic analysis and infection	Diagnosis	Disease or condition	Illness grade	ELISA result	ELISA titer	NT titer	NT result
46	34/F	6	AS	Healthy	Mild	–	0.0	<20	–
47	41/M	6	AS	Healthy	Mild	–	0.02	40	+
48	41/F	6	PN	Healthy	Moderate	–	0.76	320	+
45	42/M	6	PN	Healthy	Moderate	–	0.75	80	+
43	56/M	6	SPN	Healthy	Severe	–	3.0	80	+
44	38/F	6	SPN	Pregnant, thyroid disease	Severe	+	2.4	80	+
1	52/F	5	URTI	HPT, thyroid disease	Mild	–	0.1	<20	–
2	43/F	5	URTI	Healthy	Mild	–	0.3	42	+
15	35/M	5	PN	DM, hyperlipidemia	Moderate	B	0.8	30	+
33	39/F	4	URTI	Healthy	Mild	–	0.7	28	–
7	49/M	4	URTI	DM, HPT, BA, IHD, ESRD	Mild	+	1.5	104	+
34	42/F	4	URTI	HPT	Mild	+	1.9	144	+
40	28/F	4	URTI	Healthy	Mild	NP	NP	40	+
41	32/F	4	AS	Healthy	Mild	NP	NP	41	+
31	33/M	4	URTI	Healthy	Mild	–	0.5	34	+
32	45/F	4	URTI	Healthy	Mild	B	0.9	44	+
3	45/M	4	PN	Smoker	Moderate	+	1.1	48	+
5	61/M	4	PN	DM, HPT, IHD	Moderate	+	2.9	160	+
25	28/M	4	PN	Healthy	Moderate	+	2.5	315	+
42	47/M	4	PN	Healthy	Moderate	NP	NP	351	+
45	42/M	4	PN	Healthy	Moderate	NP	NP	162	+
29	58/M	3	URTI	Healthy	Mild	–	0.1	45	+
23	28/M	3	URTI	Healthy	Mild	–	0.1	42	+
8	47/M	3	URTI	HPT, hyperlipidemia	Mild	+	2.5	320	+
18	55/M	3	URTI	DM	Mild	+	3.4	648	+
20	34/M	3	URTI	Healthy	Mild	–	0.6	81	+
26	39/M	3	URTI	Healthy	Mild	–	0.6	75	+
35	63/M	3	URTI	DM	Mild	+	2.5	501	+
37	61/M	3	URTI	DM, HPT	Mild	+	1.2	81	+
14	32/F	3	AS	Healthy	Mild	–	0.1	45	+
39	34/M	3	URTI	Healthy	Mild	+	1.2	31	+
9	36/F	3	URTI	Healthy	Mild	–	0.2	32	+
6	74M	3	URTI	DM, lipid	Mild	–	0.1	<20	–
10	46/F	3	AS	Healthy	Mild	–	0.1	20	–
11	47/F	3	AS	Grave’s disease	Mild	–	0.1	20	–
12	33/F	3	AS	Healthy	Mild	–	0.3	20	–
17	54/F	3	URTI	HPT, thyroid disease	Mild	+	4.3	<20	–
27	29/M	3	URTI	Healthy	Mild	–	0.1	<20	–
30	41/F	3	URTI	Healthy	Mild	–	0.2	<20	–
4	41/M	3	PN	Stroke	Moderate	+	3.4	446	+
19	50/M	3	PN	Healthy	Moderate	+	3.7	315	+
24	54/M	3	PN	DM, HPT, myocarditis	Moderate	+	2.4	398	+
22	57/M	3	PN	Asthma	Moderate	+	1.9	41	+
16	62F	3	SPN	Asthma, hyperlipidemia	Severe	+	2.6	416	+
21	34/F	3	SPN	Healthy	Severe	+	2.4	375	+
28	38/M	3	SPN	Healthy	Severe	+	1.8	117	+
13	59/M	3	SPN	Healthy	Severe	–	0.1	20	–
36	64/M	2	URTI	Healthy	Mild	+	2.5	160	+
38	34/M	2	URTI	Healthy	Mild	–	0.3	27	+

*AS, asymptomatic; B, borderline; BA; bronchial asthma; DM, diabetes mellitus; ESRD, end-stage renal disease; HPT, hypertension; IHD, ischemic heart disease; NP, not performed; NT, neutralization test; PN, pneumonia; SPN, severe pneumonia (patients were in intensive care unit and required intubation and ventilation); URTI, upper respiratory tract infection; –, negative; +, positive.

The percentage of samples that had positive neutralizing antibodies was 20/28 (71.4%) at 2–3 years, 11/12 (91.7%) at 4 years, and 7/9 (77.6%) at 5–6 years postinfection (p = 0.405 for any difference and 0.349 for trend) (Table). The median (IQR) titer of neutralizing antibodies was 45 (20–319) at 2–3 years, 76 (40–162) at 4 years, and 42 (23–80) at 5–6 years postinfection (p = 0.499 for any difference and 0.755 for trend) (Figure, panel A).

**Figure:**
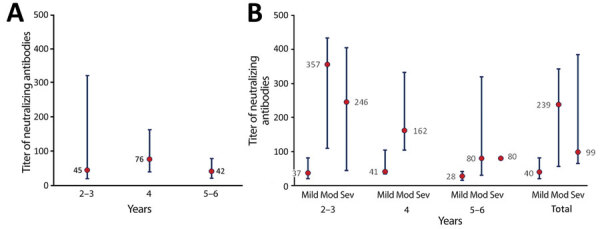
Neutralization antibody titers in Middle East respiratory syndrome (MERS) convalescent-phase serum samples measured 2–-6 years postinfection, Saudi Arabia. Three groups (patients who had mild, moderate, or severe MERS) were enrolled in this study, and serum samples were collected for neutralizing antibody detection (median focus reduction neutralization test titer) at the indicated times after recovery. The cutoff value was 1:20. Median titers of neutralizing antibody (red dots) and interquartile range (blue bars) were measured according to years postinfection (panel A) and disease severity (panel B). There was no major decrease in neutralizing antibodies over 6 years postinfection. Survivors who had moderate and severe disease had higher neutralizing antibody titers then survivors who had mild disease. Mod, moderate; Sev, severe.

Positive neutralizing antibodies were found in 21 (67.7%) of 31 survivors who had mild disease, 12 (100.0%) of 12 survivors who had moderate disease, and 5 (83.3%) of 6 survivors who had severe disease (p = 0.054 for any difference and p = 0.035 for trend) (Table). The median (IQR) titer of neutralizing antibodies was 40 (20–81) for survivors who had mild disease, 239 (56–343) for survivors who had moderate disease, and 99 (65–385) for survivors who had severe disease, respectively (p = 0.004 for any difference and p = 0.002 for trend).

Survivors who had mild, moderate, and severe disease had the following median (IQR) titers for neutralizing antibodies: 37 (20–81), 357 (110–434), and 246 (44–406) at 2–3 years postinfection (p = 0.109 for any difference and p = 0.053 for trend); 41 (34–104) and 162 (104–333) (mild or moderate disease only) at 4 years postinfection (p = 0.010); and 28 (15–42), 80 (30–320), and 80 (80–80) at 5–6 years postinfection (p = 0.130 for any difference and p = 0.065 for trend) (Figure, panel B). We found no major decrease in neutralizing antibody titers over 6 years (Figure, panel A). Survivors who had moderate and severe disease had higher titers than survivors who had mild disease over 6 years (Figure, panel B).

## Conclusions

At 6 years postinfection, we detected antibody responses in 100% of MERS survivors who had severe or moderate disease and in 50% of survivors who had mild disease, demonstrating durability of the MERS-CoV–specific antibody response. Because we did not measure MERS-CoV–specific T lymphocyte responses, the number of MERS survivors who had detectable immune responses was probably underestimated. T-cell responses were detected in several MERS survivors who had negative antibody responses at 6 months postinfection (*9*). The results are consistent with those of previous studies, which the association between disease severity and decrease of antibody response in MERS survivors over time (*10*). Similar results were described after the SARS epidemic. SARS survivors had persistent antibody responses for 3 years postinfection, and a decrease by 6 years postinfection (*11*,*12*). However, a recent study indicated that low levels of SARS-CoV–specific antibody could be detected in some survivors at 12 years postinfection (X. Guo et al., Sun Yat-sen University, pers. comm., 2020 Jan 1).

In this study, we performed ELISA and neutralizing antibody assays for all cases. Although cases of severe disease showed good concordance between the 2 assays, some cases of mild or moderate disease had a negative ELISA result and a positive neutralizing test result. Similar results were observed in camel workers who had asymptomatic MERS-CoV infections, most of whom who had negative ELISA results but detectable neutralizing antibody titers (*13*). Negative ELISA results might reflect either insensitivity of the assay or high cutoff values established by the manufacturer to minimize the rate of false-positive results. In either instance, these results suggest that negative ELISA results should be read with caution in some settings.

A limitation of our study was the small number of cases of moderate or severe disease and a lack of serial samples for nearly all patients. It will also be useful to determine whether levels of antibody would be protective if MERS-CoV reinfection occurred. In conclusion, we showed that virus-specific neutralizing antibodies are detectable in most MERS survivors for >6 years, consistent with durable immunity against the virus.

Appendix Additional information on longevity of Middle East respiratory syndrome coronavirus antibody responses in patients, Saudi Arabia.
