# Supplementary material for: Longevity of Middle East Respiratory Syndrome Coronavirus Antibody Responses in Humans, Saudi Arabia
Source: Emerg Infect Dis. 2021 May;27(5):1472–6. doi: 10.3201/eid2705.204056 (PMC8084512; doi:10.3201/eid2705.204056)
Supplement: Appendix — Additional information on longevity of Middle East respiratory syndrome coronavirus antibody responses in patients, Saudi Arabia. [file 20-4056-Techapp-s1.pdf]

Article DOI: https://doi.org/10.3201/eid2705.204056

Longevity of Middle East Respiratory Syndrome Coronavirus Antibody Responses in Humans, Saudi Arabia

Appendix

Middle East Respiratory Syndrome Coronavirus Neutralizing Antibody Assays

A Middle East respiratory syndrome coronavirus (MERS-CoV) focus reduction neutralization test was adapted from a microneutralization assay and performed in a certified Biosafety Level 3 laboratory in Guangzhou, China as described (*1*). Plasma samples (50 μL) were serially diluted, mixed with 50 μL of MERS-CoV (100 focus-forming units) in 96-well microwell plates and incubated for 1 hour at 37°C. Mixtures were then transferred to 96-well plates seeded with Vero 81 cells (American Type Culture Collection, https://www.atcc.org) for 1 hour at 37°C to enable virus entry. Inocula were then removed before adding the overlay media (100 μL minimal essential medium [MEM]) containing 1.2% carboxymethylcellulose. The plates were then incubated at 37°C for 48 hours. Overlays were removed and cells were fixed with 4% paraformaldehyde solution for 30 min. Cells were permeabilized with 0.2% Triton X-100 and incubated with a human anti-MERS-CoV-S monoclonal antibody for 1 hour at room temperature before adding horseradish peroxidase–conjugated goat anti-human IgG (heavy plus light chain) antibody (Jackson ImmunoResearch, https://www.jacksonimmuno.com). A human monoclonal antibody (tentatively named ZMC1411, diluted to 10 mg/mL) against MERS-CoV receptor binding domain was used as the primary antibody (it was isolated, cloned from memory B cells from a convalescent-phase patient, produced, and validated in our laboratory).

Cells were further incubated at room temperature. Reactions were performed with TrueBlue Peroxidase substrates (Seracare Life Sciences Inc., https://www.seracare.com). The numbers of MERS-CoV foci were calculated by using an EliSpot Reader (Cellular Technology Ltd., https://www.pharmaceutical-technology.com).

A MERS-CoV microneutralization assay was performed as described (*2*) in a certified Biosafety Level 3 laboratory in Jeddah, Saudi, because of shipment difficulties to China during

the coronavirus disease pandemic lockdown. Two-fold serial dilutions of heat-inactivated serum samples prepared in Dulbecco's modified Eagle medium starting at a 1:10 dilution were incubated with equal volumes of Dulbecco's modified Eagle medium containing 1.58×10^7 median (50%) tissue culture infectious doses/mL of MERS CoV for 1 h at 37°C in a 5% CO_2 incubator. The mixture of virus–serum was used to infect confluent Vero E6 cell monolayers in 96-well plates (in triplicate) and incubated at 37°C in a 5% CO2 incubator. Cytopathic effects were observed on a daily basis for 3 days to determine neutralizing antibody titer and stained by using crystal violet. The neutralizing antibody titer for each serum sample is reported as the reciprocal of the highest dilution that completely protected cells from cytopathic effects in 50% of the wells.

References

1.Wang Y, Zhang L, Sang L, Ye F, Ruan S, Zhong B, et al. Kinetics of viral load and antibody response in relation to COVID-19 severity. J Clin Invest. 2020;130:5235–44. PubMed https://doi.org/10.1172/JCI138759

2. Muthumani K, Falzarano D, Reuschel EL, Tingey C, Flingai S, Villarreal DO, et al. A synthetic consensus anti-spike protein DNA vaccine induces protective immunity against Middle East respiratory syndrome coronavirus in nonhuman primates. Sci Transl Med. 2015;7:301ra132. PubMed https://doi.org/10.1126/scitranslmed.aac7462
